# NIST Gas Hydrate Research Database and Web Dissemination Channel

**DOI:** 10.6028/jres.115.007

**Published:** 2010-04-01

**Authors:** K. Kroenlein, C. D. Muzny, A. Kazakov, V. V. Diky, R. D. Chirico, M. Frenkel, E. D. Sloan

**Affiliations:** Thermophysical Properties Division, National Institute of Standards and Technology, Boulder, CO 80305; Department of Chemical Engineering, Colorado School of Mines, Golden, CO 80401

**Keywords:** clathrate hydrate, database, gas hydrate, markup language, thermophysical properties, web access

## Abstract

To facilitate advances in application of technologies pertaining to gas hydrates, a freely available data resource containing experimentally derived information about those materials was developed. This work was performed by the Thermodynamic Research Center (TRC) paralleling a highly successful database of thermodynamic and transport properties of molecular pure compounds and their mixtures. Population of the gas-hydrates database required development of guided data capture (GDC) software designed to convert experimental data and metadata into a well organized electronic format, as well as a relational database schema to accommodate all types of numerical and metadata within the scope of the project. To guarantee utility for the broad gas hydrate research community, TRC worked closely with the Committee on Data for Science and Technology (CODATA) task group for Data on Natural Gas Hydrates, an international data sharing effort, in developing a gas hydrate markup language (GHML). The fruits of these efforts are disseminated through the NIST Sandard Reference Data Program [1] as the Clathrate Hydrate Physical Property Database (SRD #156). A web-based interface for this database, as well as scientific results from the Mallik 2002 Gas Hydrate Production Research Well Program [2], is deployed at http://gashydrates.nist.gov.

## 1. Introduction

The interdisciplinary field of gas hydrate research is undergoing rapid growth. Publication rates in peer-reviewed journals have displayed nearly exponential growth in the century following the discovery of hydrates in the laboratory, with more than 3000 refereed publications as of the 1990s [3]. Much of the recent growth is due to the perceived value of methane clathrate as a non-petroleum-derived large-scale energy resource [4]. Recent estimates of the world's naturally-occurring hydrated methane vary widely, ranging from 2.5 × 10^15^ m^3^ [5] to 1.2 × 10^17^ m^3^ [6] at standard temperature and pressure, but the amount of organic carbon in hydrates can be conservatively estimated as a factor of two greater than the total of all remaining petroleum and natural gas reserves [7]. The remote locations where hydrate exists and the dispersed nature of the deposits have prevented development at present, but the perceived potential has encouraged many nations, including Japan, Germany, India, China, Korea, Taiwan, Canada, and the United States to invest heavily in hydrate recovery programs.

Study of natural hydrate occurrences has shown that they typically exist close to their thermodynamic stability limit [6], so slight changes in ambient temperature or pressure may result in catastrophic release of methane, a potent greenhouse gas, with implications on global climate change [8] and seafloor slope stability [9]. Massive releases of organic carbon to the atmosphere and mass extinction events during the Permian Triassic [10], Late Jurassic [11], Late Paleocene Thermal Maximum [12], and other eras are often connected to the sudden release of hydrated gas.

Publication rates of gas hydrate information are now such that a diligent researcher could be easily overwhelmed in attempting to maintain a broad understanding of the state of the art. One solution to this difficulty is the centralization of critically evaluated data sets. Such a database can facilitate understanding of naturally occurring hydrate interactions with geophysical processes, aid in the application of hydrate knowledge to technologies involved in resource recovery and storage, and support the gas hydrate research community in general. The developed database, the scope of which includes thermophysical and structural data, provides to researchers the ability to retrieve high quality, critically evaluated data, as well as to submit new data sets. By establishing the hydrate database at the United States National Institute of Standards and Technology (NIST) in Boulder, Colorado, the viability of this project is secured well into the future. A critically evaluated hydrate database is essential for eliminating data redundancies, highlighting key data gaps, and providing an assurance of data quality to aid research efforts within the broader research community.

The data-transfer approaches associated with this data capture and storage effort are being coordinated with CODATA, which has been developing (a) a markup language called the Gas Hydrate Markup Language (GHML) [13–16] for communicating gas hydrate data throughout the research community and (b) an international hydrate portal technology for centralized access to a number of database efforts. So that the data collected by this effort will be available to such a portal, all database output is fully consistent with GHML. To aid in database access prior to the availability of such a portal, a state-of-the-art web interface was designed for the data archive. By use of a number of technologies^1^ [17–20], a native applicationlike interface rendered by use of a traditional web browser was developed. This interface provides the capability to navigate through the data sets, view them with sortable tables, chart data sets against each other, and download each data set to a local machine for additional analysis. This interface also provides access to the 2002 scientific results of the Mallik Gas Hydrate Production Research Well Program [2], reproduced with permission, and provides all capabilities of the viewing software previously developed for dissemination with physical copies of the Mallik dataset.

## 2. Data Collection and Characterization

### 2.1 General Data Collection and Characterization at TRC

This hydrate data resource development was undertaken by the Thermodynamics Research Center (TRC) [21] at NIST in Boulder, Colorado. The database at the core of nearly all TRC activities is the NIST SOURCE Data Archival System (SOURCE) [22–23], which is one of the largest relational archival experimental data systems, currently including more than 120 properties (including chemical structural information) for pure compounds, mixtures, and chemical reactions, with data records numbering in the millions. All TRC developments are built upon the algebraic constraints of the Gibbs phase rule, which specifies the number of free parameters necessary for a system to be thermodynamically well defined based on the number of chemical components and phases present. The NIST ThermoData Engine software [24–25], developed at TRC, is the first full-scale implementation of the dynamic data evaluation concept [26]. TRC also has agreements with major publishers in the field of thermophysical properties for implementation of data quality assurance (DQA) procedures at the time of data submission by authors [27]. Authors provide their manuscripts and Data Summaries (defined chemical samples, methods, properties, and uncertainties) that are used at NIST with Guided Data Capture (GDC) software [28–29] for generation of structured data files. This approach assures that submitted data are in an appropriate format [30–32] and include sufficient supporting information to allow accurate reliability estimates. In addition, the NIST ThermoData Engine software is used to check the newly submitted data for consistency with the available literature and established prediction and correlation methods. As the gas hydrate database is a critically evaluated dynamic data set, allowing for continuous updating and reliability analysis, the experience gleaned from these previous large-scale efforts was fundamental in formulating approaches to complete the present task for gas hydrates.

### 2.2 Literature Archive

A primary task of this program was the collection and characterization of a literature archive for gas hydrates. This collection had as its basis a large (3500 unique sources) electronic document archive originally assembled by Dr. E. D. Sloan. At present, the complete archive, maintained as electronic portable document format (PDF) files, contains approximately 6000 unique sources. The archive includes peer-reviewed journal articles, technical reports, master's theses, and doctoral dissertations in a number of languages with dates from the present back to the 18th century. Of the total archive, approximately 300 documents were determined to contain independent thermophysical or crystallographic data that are sufficiently well-constrained for full property specification within this project. This literature archive continues to grow as new material is published.

For the task of reviewing this article set and evaluating data content, the TRC Gas Hydrates Data Entry Facility was established in January 2008, paralleling the previously established TRC Data Entry Facility. This new group included four undergraduates in relevant technical fields from the University of Colorado at Boulder and the Colorado School of Mines. Under the direction of Dr. Kenneth Kroenlein of the TRC group, the students reviewed source materials, assembled an in-house citation database to track documents, and collected information from those files following data collection protocols established for the proven TRC Data Entry Facility. The group functioned independently until May 2009, at which time it was folded into the TRC Data Entry Facility as the data processing operation transitioned into a maintenance phase. The group continues to analyze newly obtained materials and will continue to add them to the database as appropriate.

### 2.3. Guided Data Capture

Information from original data sources is not entered directly into the NIST SOURCE Data Archival System (SOURCE) but is captured or “compiled” in the form of batch data files (coded ASCII text). This allows application of extensive completeness and consistency checks during the capture process before the data are loaded into the central repository. Due to the complexity of the properties and chemical systems involved, extensive expertise has traditionally been required for data compilation. Moreover, expertise in data and measurements is needed to assess uncertainties for each property value. In establishment of the Data Entry Facility at NIST, two major concerns were identified: (1) how to ensure quality of captured information with technically sound but inexperienced data compilers and (2) how to minimize errors before the data are introduced into SOURCE. To meet these goals, interactive Guided Data Capture (GDC) software, written in Microsoft Visual Basic, was developed. The program guides data capture and provides convenient review and editing mechanisms. Undergraduate students involved in in-house data capture played, and continue to play, a key role in development and testing of the GDC software.

GDC functions to guide inexperienced but technically competent individuals through the process of extracting information from the literature, ensuring completeness, validating the information through data definition, range checks, etc., and guiding initial uncertainty assessment to ensure consistency between compilers with diverse levels of experience. A key feature of the GDC software is capturing of information in close accord with customary original-document formats and leaving transformation to formalized data records within the scope of the software procedures. Thus, GDC relieves the compiler of the need for knowledge related to the structure of the SOURCE data system, thereby eliminating common errors related to data types, length, letter case, and allowable codes. The users of GDC are scientists or students in a science or engineering discipline with varying levels of experience, but with competence in the fields of chemistry and chemical engineering.

The GDC program was developed to serve as a powerful and comprehensive tool for in-house data capture operations, as well as a data-collection and transformation aid for authors of scientific and engineering publications. The original software, without support for gas hydrate property capture, is available for free downloading via the World Wide Web [29]. Comprehensive documentation for the software is included. The GDC software has features that allow ready detection of inconsistencies and errors in reported data (erroneous compound identifications, typographical errors, etc.), resulting in improved integrity of the captured data over that given in the original sources. Additional information on the development of GDC can be found in the literature [28].

In order to capture experimental data sets pertaining to samples of gas hydrate, the existing GDC software required significant modification. Whereas data normally processed through GDC are either for a pure compound or a mixture of a small number of well-defined compounds in well-defined ratios, a gas hydrate is a non-stoichiometric structure, where chemical composition may be undetermined, but which can still yield valuable data. Whereas it might be desirable to simply dismiss such studies as unreliable, the comparative paucity of data precludes such a determination. The solution to this conflict was determined to be the creation of an original data structure within the GDC framework that behaves in many ways like a new compound, defined by the combination of its constituents and known thermodynamic properties. With these modifications, the GDC software supports the capture and organization of data pertaining to bulk properties (e.g., mass specific volume, thermal conductivity, heat capacity at constant pressure per unit mass, speed of sound), phase equilibrium with an arbitrary number of components and phases, crystalline structure and enthalpy of hydrate decomposition for gas hydrates. In particular, the data format for crystalline structure represents an entirely new development within this software. The level of functionality thus attained represents significant progress towards a complete GDC software package for gas-hydrate data.

**Fig. 1:**
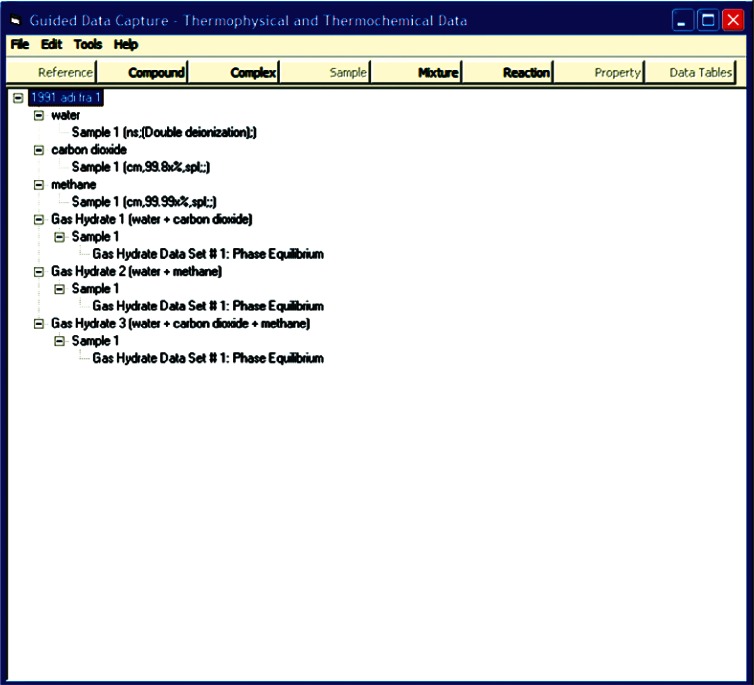
Screen capture of tree structure for a gas hydrate sample characterization within GDC. Example shown is for carbon dioxide + water, methane + water and carbon dioxide + methane + water systems for hydrate + aqueous + vapor equilibria, extracted from Ref. [35].

**Fig. 2:**
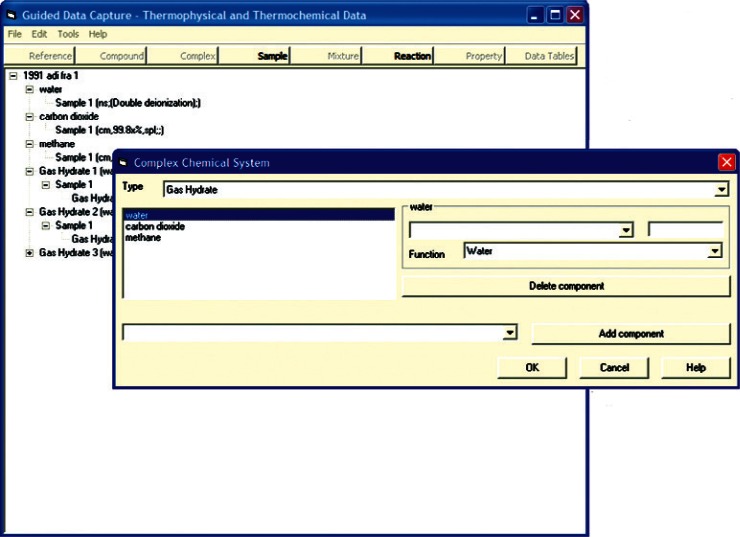
Screen capture of GDC dialog for definition of a gas hydrate system for carbon dioxide + methane + water, extracted from Ref. [35].

**Fig. 3:**
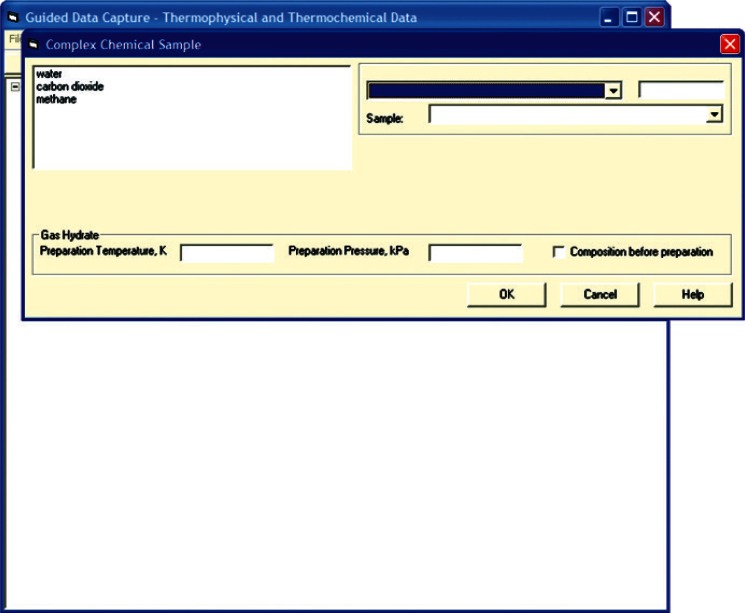
Screen capture of GDC dialog for definition of a gas hydrate sample for carbon dioxide + methane + water, extracted from Ref. [35].

The basic tree structure of GDC data (Fig. 1) is organized around that of the data source document. Following from that are definitions of chemical components in the systems presented within the citation and specific sample information with detailed purity information. A gas hydrate system is then defined by a combination of those chemical components (Fig. 2) and a gas hydrate sample is defined through the association of specific samples of those components, as well as the conditions under which the hydrate was formed, if appropriate (Fig. 3). It is only when all of this detailed information regarding purity of constituent compounds is defined that measured properties are entered, allowing for a better understanding of the resultant data reliability.

**Fig. 4:**
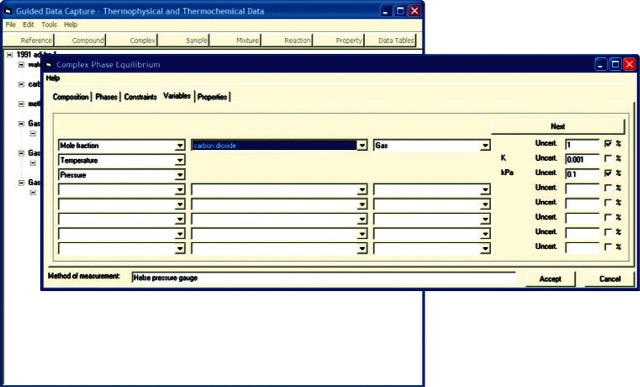
Screen capture of GDC dialog for defining phase equilibrium constraints and variables on a given set of phase equilibrium data for carbon dioxide + methane + water, extracted from Ref. [35].

**Fig. 5:**
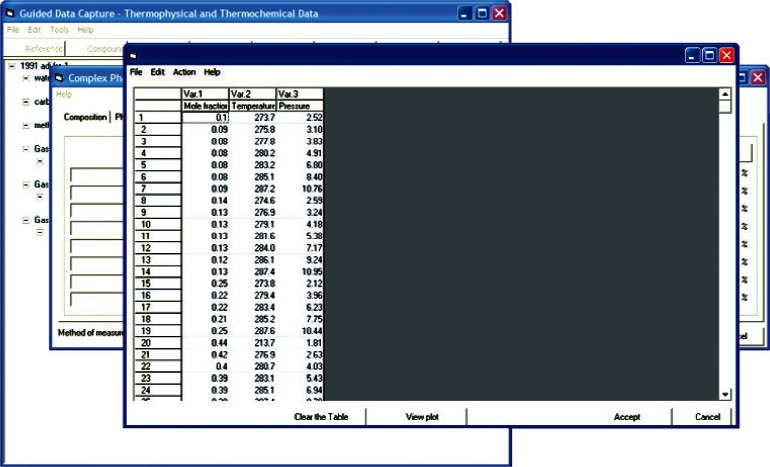
Screen capture of GDC dialog for entering tabulated data associated with hydrate + aqueous + vapor phase equilibrium data for carbon dioxide + methane + water, extracted from Ref. [35].

**Fig. 6:**
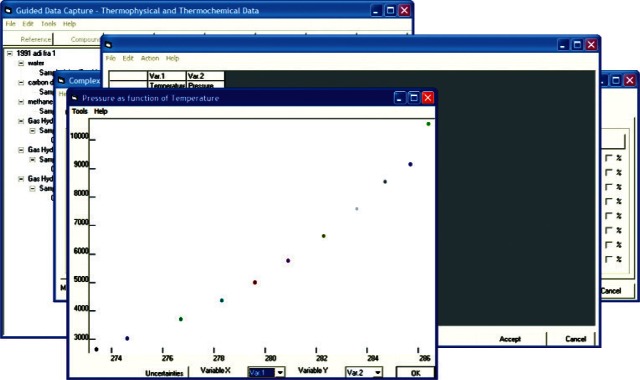
Screen capture of natively-generated graph of data entered into GDC tabulated data dialog for pressure as a function of temperature for hydrate + aqueous + vapor phase equilibrium in methane + water system, extracted from Ref. [35].

In order to ensure a well defined thermodynamic state and to prevent storage of dependent variables as independent, the system is constrained according to the Gibbs Phase Rule. For example, if a three-phase region is being defined in a gas hydrate sample formed from three guest molecules (Fig. 4), there exist two degrees of freedom in the system, and hence, two independent variables are required to define the system. Any additional data values at a point are then dependant variables and original information about the system. The data for the system are then recorded in an internal data table (Fig. 5). To prevent transcription errors on the part of the data entry technician, data are copied directly from electronic versions of the source, either obtained via electronic distribution or via text recognition software applied to digitized material. Data consistency can then be verified by use of native graphing capabilities (Fig. 6) within the GDC software.

**Fig. 7:**
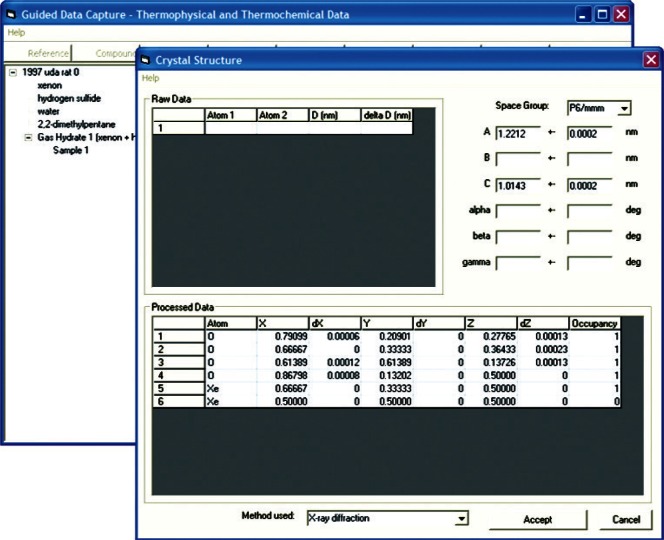
Screen capture of GDC dialog for storing crystallographic data, including space group, unit cell parameters and atom distribution for xenon + hydrogen sulfide + 2,2-dimethylpentane + water system (structure H), extracted from [36].

Clathrate hydrates primarily occur in one of three crystalline structures (termed sI, sII and sH), although a number of more exotic configurations are known. The conformation assumed by a hydrate is primarily a function of the guest molecules, and is the most significant factor in determining hydrate stability boundaries. Characterizing crystal structure is a wholly novel addition to GDC intended for gas hydrate data collection. In order to maintain future extensibility, as well as to collect detailed information about the hydrate cage structure, information is stored regarding the crystallographic space group, unit cell dimensions, and both raw and processed information regarding the atom distribution (Fig. 7). This new data structure was modeled upon the Crystallographic Information File (CIF) data file format. CIF is an International Union of Crystallography (IUCr) standard used within the crystallographic community for communication of experimental results [33].

### 2.4. Database Architecture

Establishment of a comprehensive data depository is one of the major challenges in implementation of the dynamic data evaluation concept. The NIST SOURCE Data Archival System [22–23] was designed and built to be such a depository for experimental thermophysical and thermochemical properties for organic compounds reported in the world's scientific literature. The scope of the data system includes more than one hundred defined properties for pure compounds, binary and ternary mixtures, and reacting systems. SOURCE now contains nearly four million numerical values for this range of systems.

**Fig. 8:**
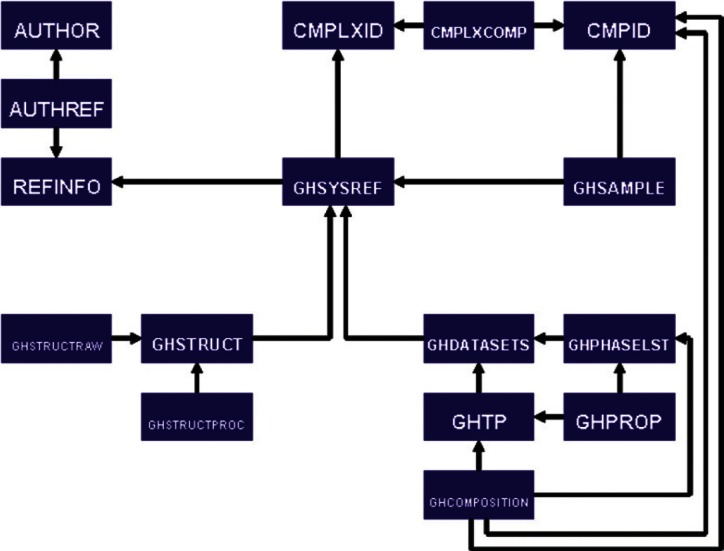
Schematic representation of new SOURCE table structure and gas-hydrate-relevant table substructure.

**Fig. 9:**
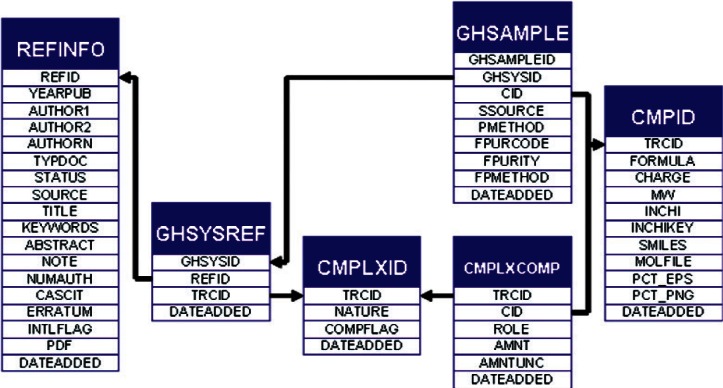
SOURCE tables relevant to defining a specific gas hydrate sample, dependant upon the literature source of the data, the chemical compounds present and the compositional purity of the feed materials.

In designing data structures to accommodate the gas hydrate data sets, limitations of the existing SOURCE architecture associated with the definitions of complex materials became apparent. In order to support these new relationships, as well as those of ionic liquids, stereo-isomeric mixtures, and other complex samples, a new table structure was designed. The relationships for the total gas hydrate system are shown broadly in Fig. 8, and the specific details required to define a complex are shown in Fig. 9. All gas-hydrate-specific tables are denoted by the "GH" prefix. To define a chemical complex (table CMPLXID), a series of well-defined compounds (table CMPID) is associated with compositional information, if appropriate, through a pivot table (table CMPLXCOMP). Each complex is assigned an identifier that is unique between the CMPID and CMPLXID tables. This allows property data to be defined equivalently in either case, independent of whether it is associated with a pure compound or a complex system. A gas hydrate complex entry is then associated with the literature source of its data through the unmodified, previously existing literature reference tables in the GHSYSREF table. As purity information of the feedstock is relevant to the ultimate properties of a crystal sample, that information is tied to the system for each component through a gas-hydrate-specific GHSAMPLE table.

**Fig. 10:**
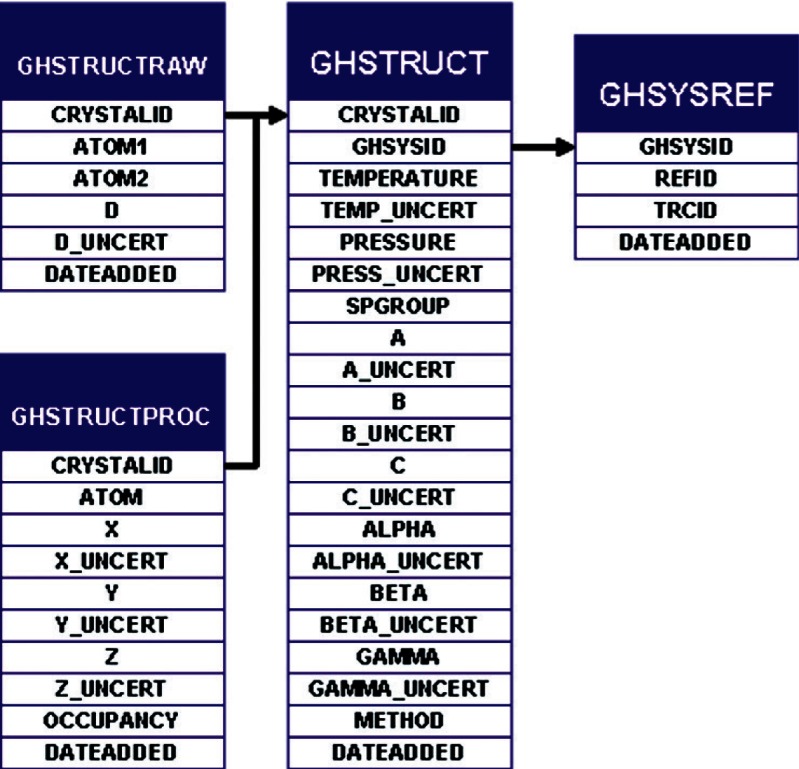
SOURCE tables relevant to defining data from crystallographic studies, including atomic distribution if reported.

If a study is crystallographic in nature, a table entry is made in table GHSTRUCT (Fig. 10). This contains basic crystallographic data (space group, lattice parameters) in addition to experimental conditions (system temperature, system pressure, uncertainty in lattice parameters, methodology). If the interatomic spacing or Cartesian atomic distribution within the unit cell is reported, such information is stored in tables GHSTRUCTRAW or GHSTRUCTPROC, respectively. This data structure follows that of a Crystallographic Information File (CIF).

Characterization of the complex phase equilibria for gas hydrates, necessary to properly specify the conditions of a thermophysical measurement, required significant extension to the existing SOURCE data storage format. Given that a gas hydrate system may contain from two to an arbitrarily large number of chemical components, a fixed table width, previously utilized to guarantee proper system constraint, becomes untenable with a gas-hydrate system. This is demonstrated readily with the application of the Gibbs phase rule to a hydrate-forming natural gas system in equilibrium with sea-water. For an eight-component representation of sea water and an eight component natural gas, this three-phase condition would require fifteen data values for proper constraint. Designing a single table to accommodate this set, in addition to data sets containing two compounds and four phases, would be highly inefficient and inherently limited, if a more complex set were encountered subsequently.

**Fig. 11:**
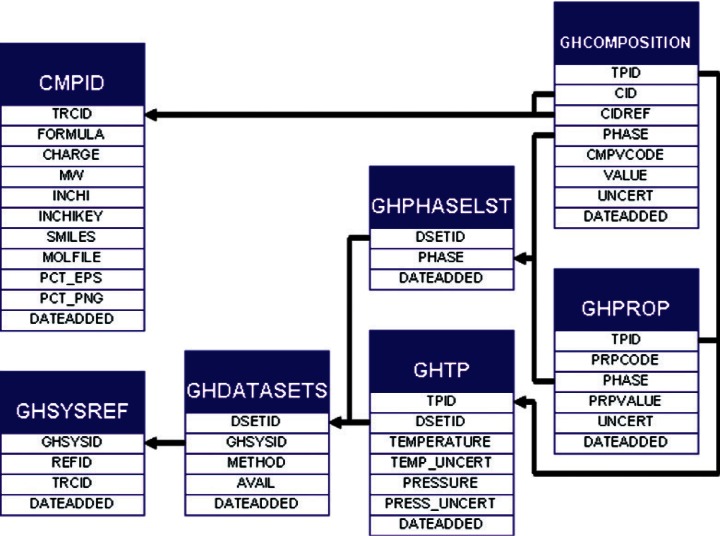
SOURCE tables relevant for defining thermodynamic state and property data, including temperature, pressure and compositional information.

The solution adopted here is shown in Fig. 11. Each data series from a given study, defined to be a set of measurements performed by one experimental method on a system with a prescribed set of phases present, is uniquely defined in the GHDATASETS table. Observed phases for this data set are stored in the GHPHASELST table. As nearly all data points have temperature and pressure values associated with them, the primary key for a given data point is specified in the GHTP table. Any additional compositional information for that point is stored in the GHCOMPOSITION table, which stores not only the composition values and uncertainties, but also the identity of the compound measured and the associated phase. Data integrity for those composition data is checked by ensuring that the referenced phases and compounds are present in the system and GHSAMPLE data provided. Property data sets, such as speeds of sound or heat capacities, are stored in the GHPROP table with similar constraints. The number of compounds, phases, and data values can then be compared and the thermodynamic completeness of a set, as expressed by the Gibbs phase rule, can be determined.

All GDC output files generated by data compilers in the Gas Hydrates Data Entry Facility are uploaded into the SOURCE archive after each file is checked for consistency with the original source material by senior staff. When appropriate validating data are available, new results are verified against stored values, providing integrity checks on historic data, as well as providing verification for new data. At present, the database contains about 12,000 individual experimental data points for about 150 compounds spanning 400 different chemical systems.

## 3. Data Dissemination Channels

### 3.1 Gas Hydrate Markup Language

A thermodynamic property data archive represents a key foundation for development and improvement of all chemical process technologies. However, rapid growth in the number of custom-designed software tools for engineering applications has created an interoperability problem between the formats and structures of thermodynamic data files and required input/output structures for the software applications. Establishment of efficient means for thermodynamic data communications is critical for provision of solutions to such technological challenges as elimination of data processing redundancies, creation of comprehensive data archives, and rapid data propagation from measurement to data management system and from data management system to engineering application. Taking into account the diversity of thermodynamic data and the numerous methods of their reporting an d presentation, it is apparent that standardization of thermodynamic data communications is a complex task.

A component of the work performed consisted of reconciling the GHMLv1.0 schema [13–16], an XML format developed prior to this project for communication of gas hydrate data, and ThermoML [30–32], the IUPAC standard for experimental and critically evaluated thermodynamic property data communication and storage. The structure of ThermoML is based on rational storage of property data with the origin of the data as a major component of the organization construct. The early efforts in the development of GHML attempted to at most only minimally disturb the published data structure and maintained a series of parallel (i.e., non-intersecting) sections that described various types of property data ("field" [14], "laboratory" [15], and "modeling" [16]). Consistency with ThermoML was effected primarily via modification of the laboratory section and the addition of citation information. Given the general inconsistency in style and nomenclature across the different sections present in the initial schema, an effort in reformulating the laboratory section was put toward rectifying this disparity. As development of GHML is an international effort under the auspices of the International Council for Science's Committee on Data for Science and Technology (CODATA), any proposed schema must be approved by that body. The rectified "laboratory" section was approved at a meeting of the CODATA Hydrate Database Steering Committee on October 27, 2007, with the note that additional unification across the disparate GHML branches was desirable.

**Fig. 12:**
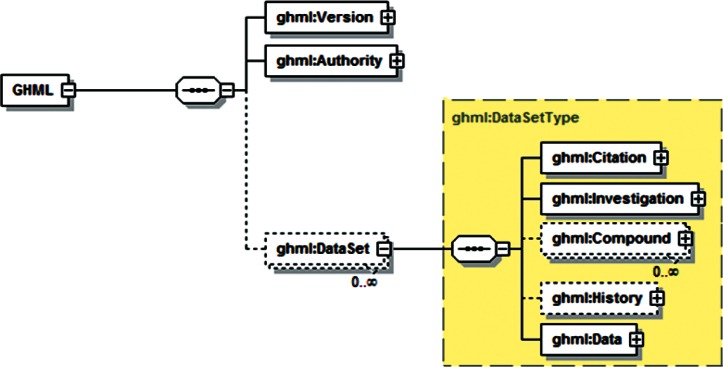
Root element of GHML.

**Fig. 13:**
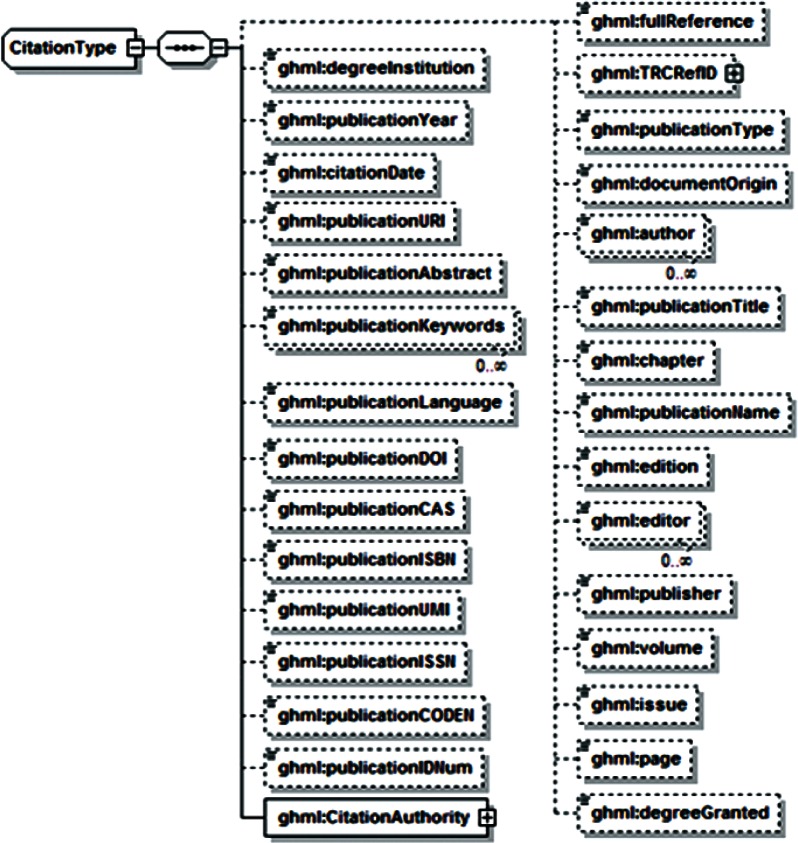
GHML citation element, consistent with ThermoML.

**Fig. 14:**
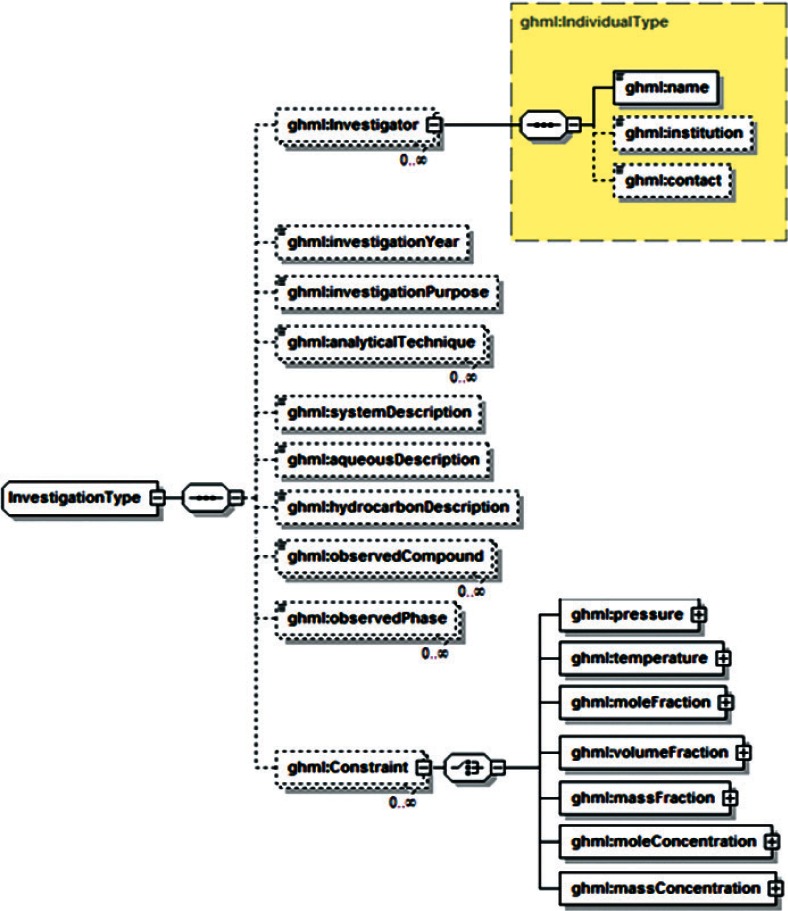
GHML investigation element.

**Fig. 15:**
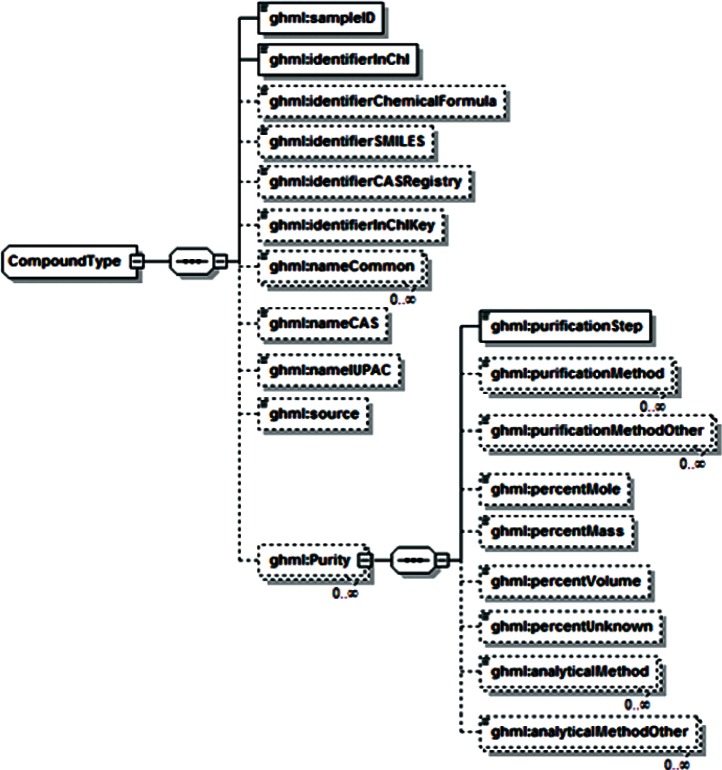
GHML compound element.

**Fig. 16:**
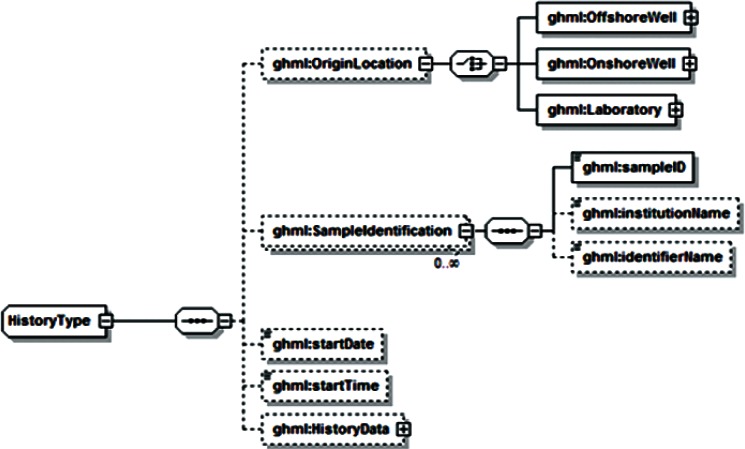
GHML history element.

**Fig. 17:**
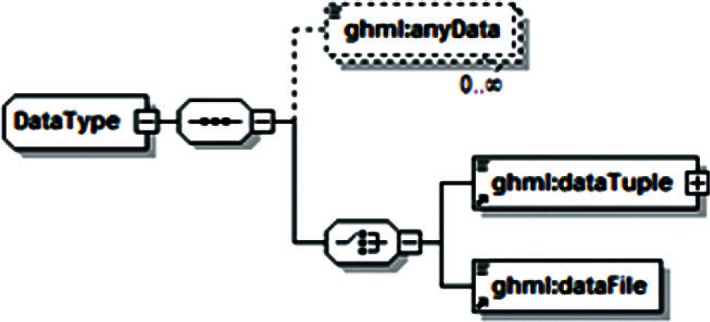
GHML data element.

**Fig. 18:**
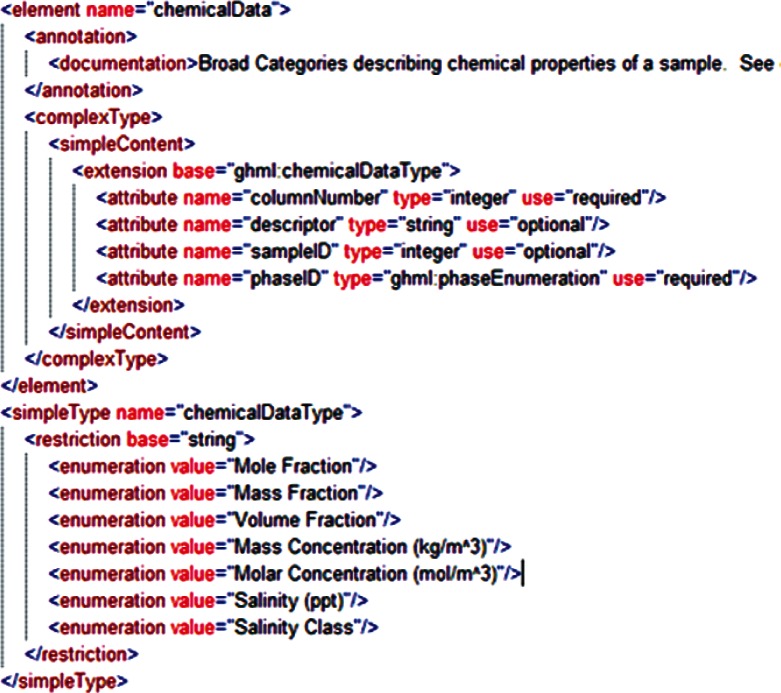
Sample data category from GHML, specifically the ChemicalData subtype.

Examination of additional data sets across the range of disciplines associated with gas-hydrate studies revealed a range of data that was unsupported by both GHMLv1.0 and the newly revised version, and it was not clear that these data could be represented by reasonable extensions to either format. For example, particle distribution studies performed by Medioli for the 2002 Mallik research well [2] would have required creating new data fields for all but one of the reported sets, and repetition of this redesign process would have been required for most new data sets. In response, a significant modification of GHML was formulated which combined the FieldData, LabData and ModelData elements into a single DataSet element (Fig. 12). Rather than specifying the structure of datasets to be encoded within the XML Schema Definition (XSD), this DataSet element specifies the encoding for metadata common to many different datasets, in the broad categories literature citation (Fig. 13), investigation details (Fig. 14), chemical compound information (Fig. 15), and sample history (Fig. 16), and then specifies the data organization of a formatted data-tuple (doubly-delimited list) through the inclusion of data labels that include appropriate attributes to maintain data relationships (Fig. 17); for example, a mole fraction data series includes relational information to specify a compound being measured and its associated phase (Fig. 18). This development was discussed at the 6th International Conference on Gas Hydrates in 2008 [34].

The 2008 revision of GHML is being used as the basis for the current web-dissemination technology development efforts underway by the CODATA Hydrate Database Task Group. It is expected that by providing a uniform model for data and metadata communication for the gas hydrates community, communication across the varied disciplines associated with these studies can be improved and scientific progress in the field can be facilitated.

### 3.2 World Wide Web-based Data Dissemination

A web-based interface specific to the Clathrate Hydrate Physical Property Database was developed to guarantee free and open access to the data resources upon completion of primary development, independent of CODATA progress. The basis for the web interface is Google Web Toolkit [17], an open source set of tools for generation of complex JavaScript front-end applications from Java source code. This set of tools was used to couple server-side database interaction to a clientside user interface built with the Ext JS [18] graphical JavaScript libraries and coupled together with open-source GWT-Ext libraries [19].

**Fig. 19:**
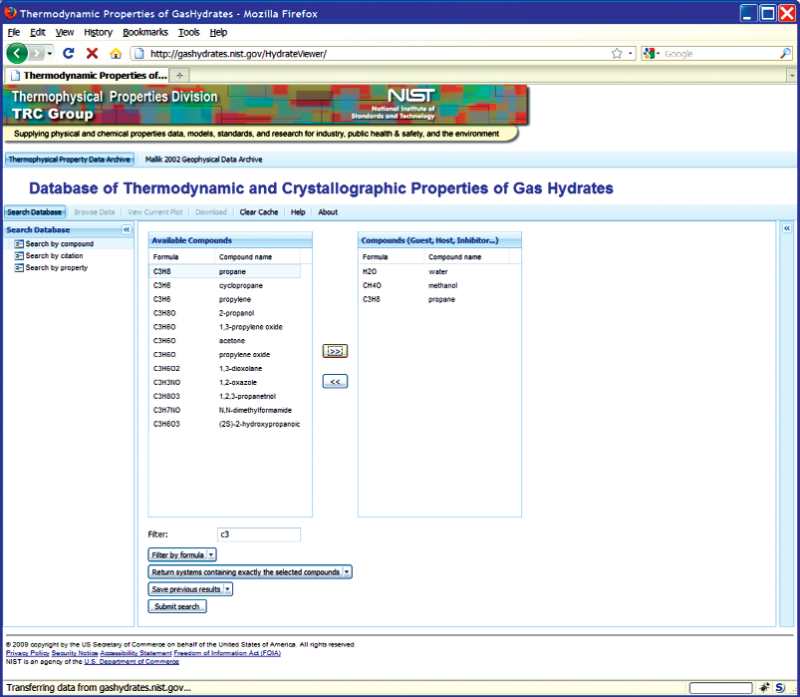
Screenshot from web interface, demonstrating a search for a chemical system, including compound filtering.

**Fig. 20:**
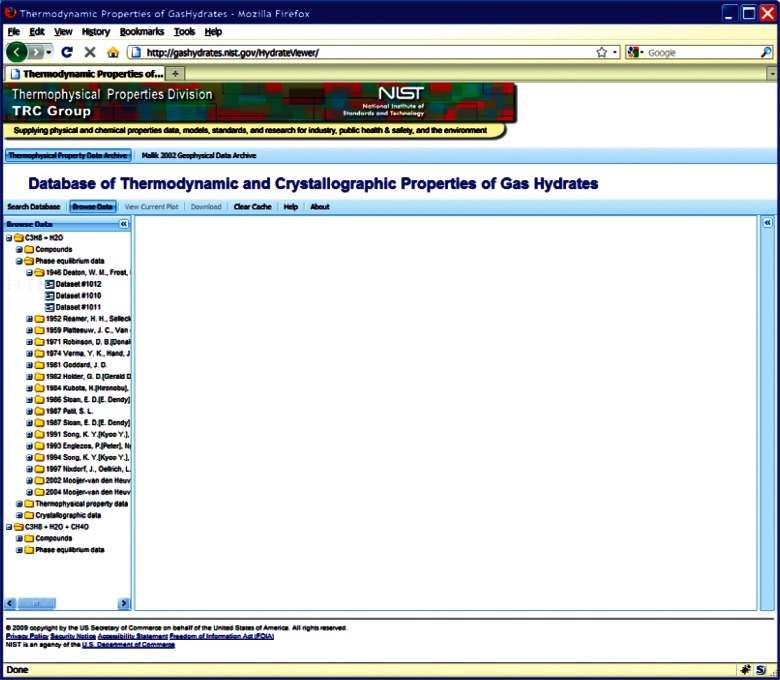
Screenshot from web interface, demonstrating search results for chemical system including dynamically populated tree..

**Fig. 21:**
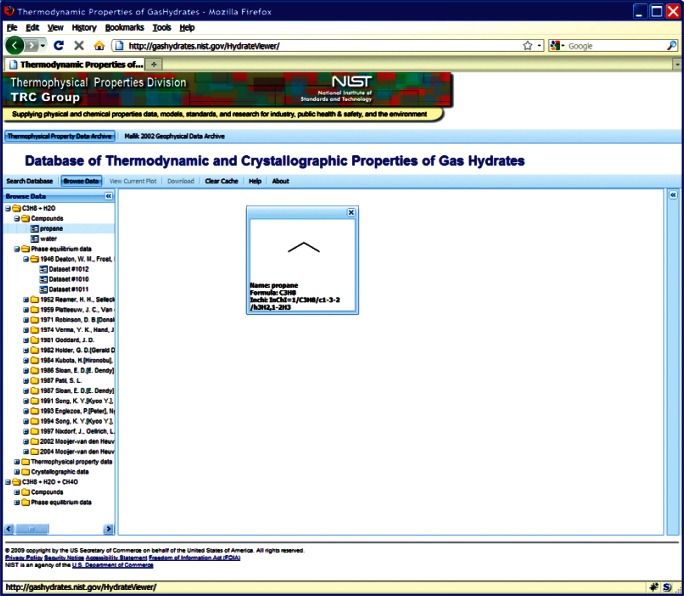
Screenshot from web interface, demonstrating display of compound information, associated with a given chemical system..

**Fig. 22:**
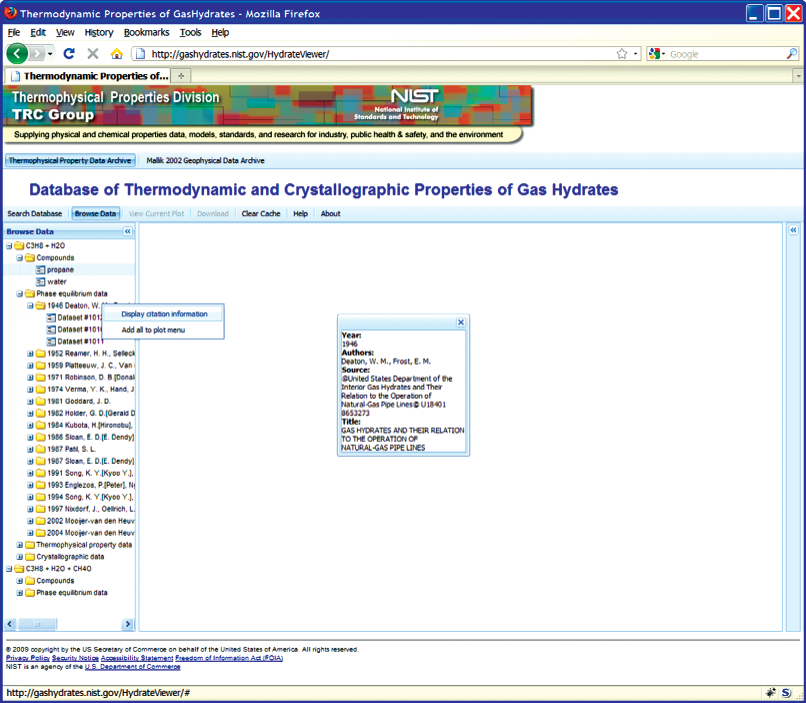
Screenshot from web interface, demonstrating display of full citation information for a given data set.

**Fig. 23:**
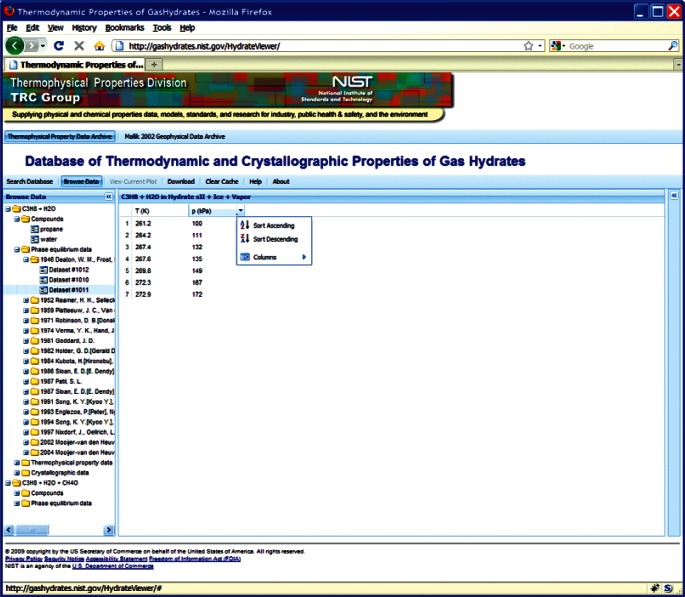
Screenshot from web interface, demonstrating tabular display of data, including sorting capability.

**Fig. 24:**
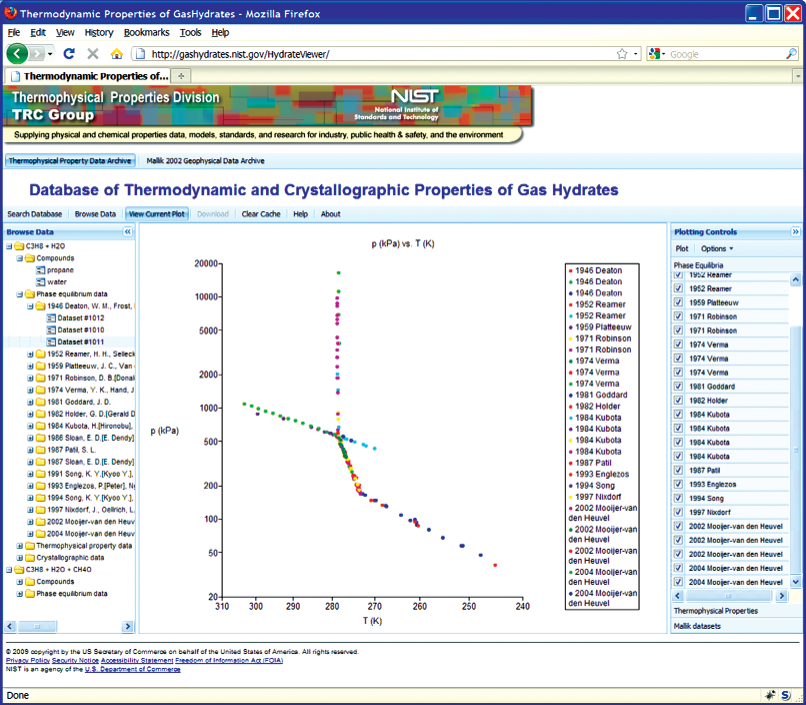
Screenshot from web interface, demonstrating Arrhenius plot of propane + water phase equilibria.

**Fig. 25:**
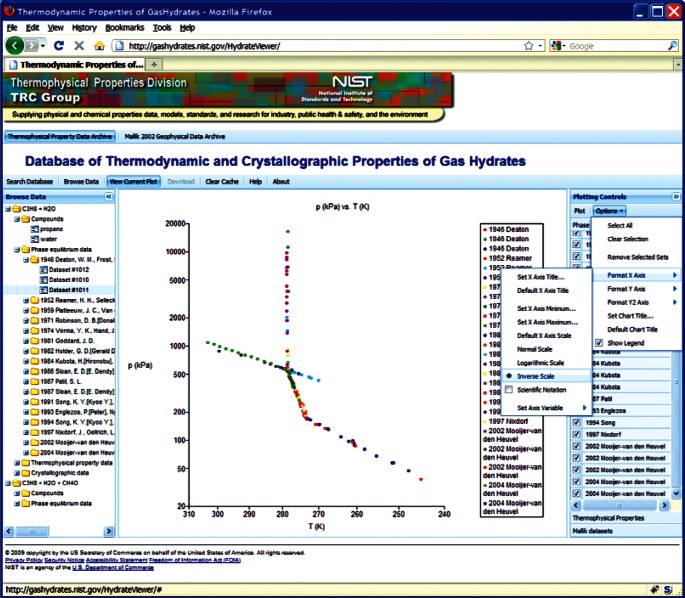
Screenshot from web interface, demonstrating chart options menu for native charting capability.

**Fig. 26:**
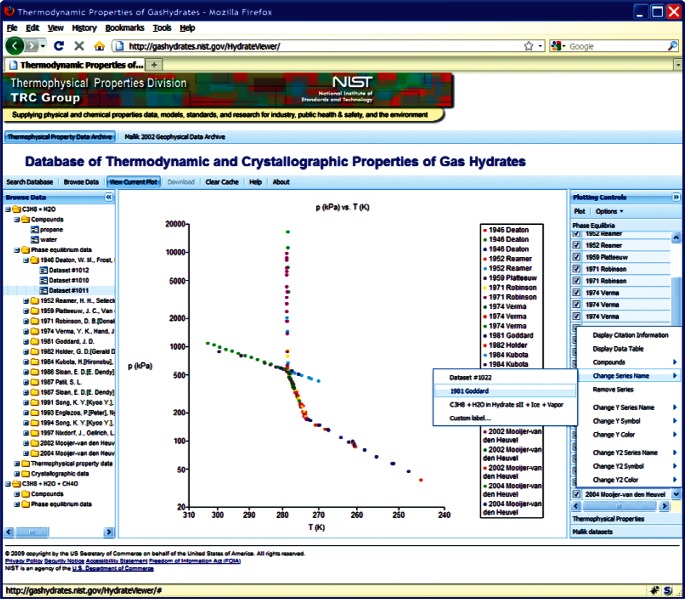
Screenshot from web interface, demonstrating dataset options menu for native charting capability.

**Fig. 27:**
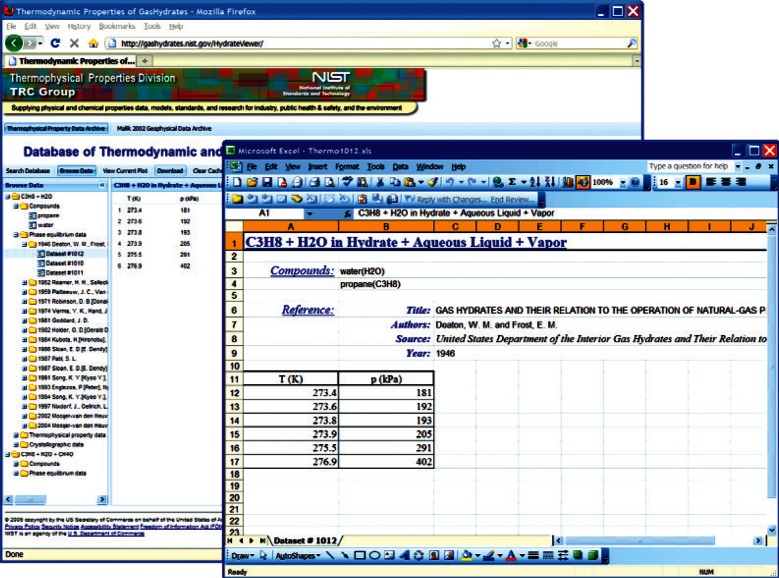
Screenshot from web interface, demonstrating downloaded table file with original table within web viewer context.

This choice of technologies allows for an interactive user experience more akin to a desktop application than with traditional web technologies. Traditional web-based database interfaces support simple search and display capabilities and require additional network transactions to modify data display. In contrast, the advanced technologies underpinning this web interface allow significantly more complex user interactions without an associated increase in required network traffic, usually the slowest step in any World Wide Web interaction. For example, the web interface supports complex queries regarding combinations of chemical compounds through dynamically populated and readily searchable lists of chemical compounds (Fig. 19). Once a user has specified search criteria, the client interface downloads the associated datasets to the client and no further server interactions are necessary. The retrieved datasets dynamically populate a tree based structure (Fig. 20), based upon chemical composition and literature source. The user can see information on the compounds involved in the systems of interest, including a two-dimensional structural representation (Fig. 21) and full citation information (Fig. 22). The user can display the datasets of interest in interactive tables (Fig. 23) that allow the user to sort data as desired. All data can be displayed graphically (Fig. 24), and the user can control axis scale, select logarithmic or inverse scaling (Fig. 25), and re-label datasets or chart objects as desired (Fig. 26). Finally, all datasets can be downloaded in a commonly accessible format (Fig. 27) for off-line processing by the user.

**Fig. 28:**
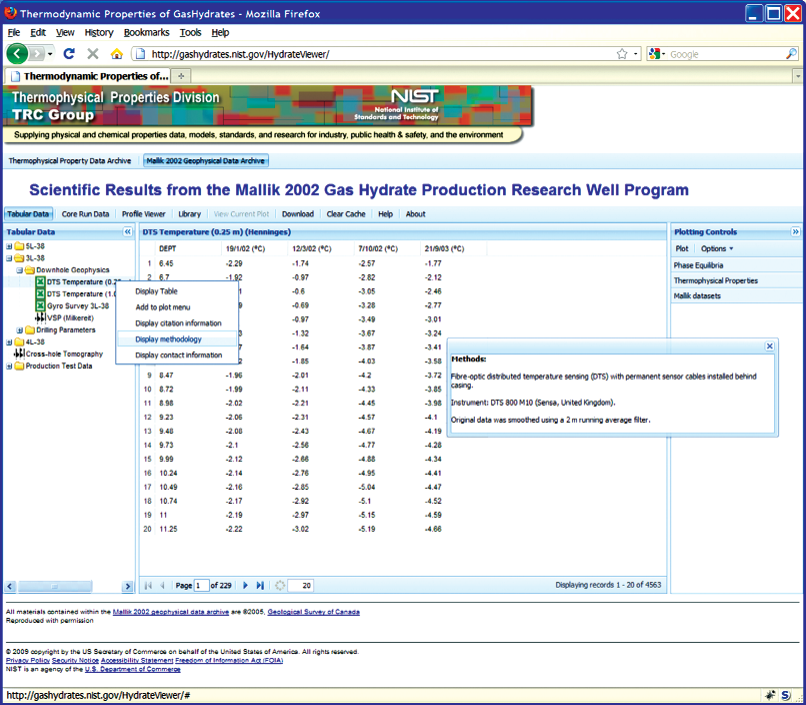
Screenshot from web interface, demonstrating table from 2002 Mallik scientific results with data methodology information displayed.

In addition to providing access to the archive of clathrate hydrate physical properties, users can also freely access the 2002 scientific results from the Mallik Gas Hydrate Production Research Well Program [2], a novel exploration of the technical feasibility of natural gas production from a permafrost-based deposit of gas hydrates. These data are reproduced with explicit, written consent of the copyright holder (Natural Resources Canada). The web-based interface offers all interactive capabilities and access to all data available through the software distributed with the original materials without the need to install software locally or permanently download materials. This includes access to all metadata, as well as original tabulated data files (Fig. 28).

## 4. Summary

The NIST Thermodynamics Research Center has completed design, population, and publication to the World Wide Web of the Clathrate Hydrate Physical Property Database (NIST Standard Reference Database 156). This data resource is available on a free and open basis at the URL http://gashydrates.nist.gov. It contains well defined and critically evaluated experimentally derived thermophysical and structural data for clathrate hydrates, including the complex systems associated with clathrate hydrates of natural gas. At present, the database contains about 12,000 individual data points for about 150 compounds spanning 400 different chemical systems. The design of this database is derived from the structure of the SOURCE Data Archive and includes novel extensions to represent complex gas hydrate data sets in a well constrained manner. This interface also provides access to the 2002 scientific results of the Mallik Gas Hydrate Production Research Well Program, reproduced with permission, and provides all capabilities of the viewing software previously developed for dissemination with physical copies of the project results.

Population of the Clathrate Hydrate Physical Property Database was supported by a gas hydrates library collected by TRC staff. Presently at nearly 6000 documents, this literature archive will continue to grow as new materials become available and will continue to serve as a source of new results to be added to the database. The literature archive was cataloged by use of an internal database and experimental datasets were extracted by use of specially designed Guided Data Capture software, facilitating data collection by individuals who are technically competent but lack the broad experience usually required to dissect complex thermophysical systems.

The original version of the gas hydrate markup language (GHMLv1.0) was modified significantly to meet the needs of the broadly based gas hydrate community. The range of data sets that can be represented was increased, support for citation information was added, and consistency with the IUPAC-standard ThermoML was included. This new GHML data format is now being used in international data-sharing development efforts. It is expected that in the future, GHML will serve as a major tool for data exchange across the boundaries of traditional academic disciplines within the gas hydrate community.

It is expected that the Clathrate Hydrate Physical Property Database, available on a free and open basis and accessible through the World Wide Web, will facilitate research on and development of technologies relevant to clathrate hydrates by providing reliable, critically evaluated data sets from the scientific literature and an intuitive interface for visualizing and comparing those data sets against one another.

## Footnotes

1 Certain commercial equipment, instruments, or materials are identified in this paper to foster understanding. Such identification does not imply recommendation or endorsement by the National Institute of Standards and Technology, nor does it imply that the materials or equipment identified are necessarily the best available for the purpose.

## References

[1] NIST Standard Reference Data Program.

[2] Dallimore SR, Medioli BE, Laframboise RR, Giroux D, Dallimore SR, Collett TS (2005). Mallik 2002 Gas Hydrate Production Research Well Program: Well Data and Interactive Data Viewer; Appendix A in Scientific Results from the Mallik 2002 Gas Hydrate Production Research Well Program, Mackenzie Delta, Northwest Territories, Canada. Gelogical Survey of Canada, Bulletin 585.

[3] Sloan ED (2004). Introductory overview: Hydrate knowledge development. American Mineralogist.

[4] (2000). Methane Hydrate Research and Development Act of 2000, 30 United States Code 1902 note. Public Law.

[5] Milkov AV (2004). Global estimates of hydrate-bound gas in marine sediments: how much is really out there?. Earth-Science Reviews.

[6] Klauda JB, Sandler SI (2005). Global Distribution of Methane Hydrate in Ocean Sediment. Energy & Fuels.

[7] Radler M (2000). World crude and natural gas reserves rebound in 2000. Oil & Gas Journal.

[8] Reagan MT, Moridis GJ (2007). Oceanic gas hydrate instability and dissociation under climate change scenarios. Geophysical Research Letters.

[9] Kvenvolden KA (1999). Potential Effects of Gas Hydrate on Human Welfare. Proceedings of the National Academy of Sciences of the United States of America.

[10] Berner RA (2002). Examination of Hypotheses for the Permo-Triassic Boundary Extinction by Carbon Cycle Modeling. Proceedings of the National Academy of Sciences of the United States of America.

[11] Padden M, Weissert H, de Rafelis M (2001). Evidence for Late Jurassic release of methane from gas hydrate. Geology.

[12] Dickens GR (2001). Carbon addition and removal during the Late Palaeocene Thermal Maximum: basic theory with a preliminary treatment of the isotope record at ODP Site 1051. Blake Nose, Geological Society, London, Special Publications.

[13] Sloan D, Kuznetsov F, Lal K, Loewner R, Makogon Y, Moridis G, Ripmeester J, Royer J, Smith T, Tohidi B, Uchida T, Wang J, Wang W, Xiao Y (2007). A Hydrate Database: Vital to the Technical Community. Data Science Journal.

[14] Löwner R, Cherkashov G, Pecher I, Makogon YF (2007). Field Data and the Gas Hydrate Markup Language. Data Science Journal.

[15] Smith T, Ripmeester J, Sloan D, Uchida T (2007). Gas Hydrate Markup Language: Laboratory Data. Data Science Journal.

[16] Wang W, Moridis G, Wang R, Xiao Y, Li J (2007). Modeling Hydrates and the Gas Hydrate Markup Language. Data Science Journal.

[17] Google Web Toolkit—Google Code.

[18] Ext—A foundation you can build on.

[19] GWT—Ext Widget Library.

[20] GChart—Google Code.

[21] TRC Group—NIST Thermophysical Properties Division.

[22] Frenkel M, Dong Q, Wilhoit RC, Hall KR (2001). TRC SOURCE Database: A Unique Tool for Automatic Production of Data Compilations. International Journal of Thermophysics.

[23] Yan X, Dong Q, Frenkel M, Hall KR (2001). Window-Based Applications of TRC Databases: Structure and Internet Distribution. International Journal of Thermophysics.

[24] Frenkel M, Chirico RD, Diky V, Yan X, Dong Q, Muzny C (2005). ThermoData Engine (TDE): Software Implementation of the Dynamic Data Evaluation Concept. Journal of Chemical Information and Modeling.

[25] ThermoData Engine http://www.trc.nist.gov/tde.html.

[26] Wilhoit RC, Marsh KN (1999). Future Directions for Data Compilations. International Journal of Thermophysics.

[27] Cummings PT, de Loos T, O’Connell JP, Haynes WM, Friend DG, Mandelis A, Marsh KN, Brown PL, Chirico RD, Goodwin ARH, Wu J, Weir RD, Trusler JPM, Pádua A, Rives V, Schick C, Vyazovkin S, Hansen LD (2009). Joint Statement of Editors of Journals Publishing Thermophysical Property Data, Process for Article Submission for The Journal of Chemical Thermodynamics, Fluid Phase Equilibria. International Journal of Thermophysics, Thermochimica Acta, and Journal of Chemical Engineering Data, Fluid Phase Equilibria.

[28] Diky VV, Chirico RD, Wilhoit RC, Dong Q, Frenkel M (2003). Windows-Based Guided Data Capture Software for Mass-Scale Thermophysical and Thermochemical Property Data Collection. Journal of Chemical Information and Computer Science.

[29] Guided Data Capture http://www.trc.nist.gov/GDC.html.

[30] Frenkel M, Chirico RD, Diky VV, Dong Q, Marsh KN, Dymond JH, Wakeham WA, Stein SE, Königsberger E, Goodwin ARH (2006). XML-Based IUPAC Standard for Experimental, Predicted, and Critically Evaluated Thermodynamic Property Data Storage and Capture (ThermoML). Pure and Applied Chemistry.

[31] International Union of Pure and Applied Chemistry.

[32] ThermoML Archive http://www.trc.nist.gov/ThermoML.html.

[33] Hall SR, Allen FH, Brown ID (1991). The Crystallographic Information File (CIF)—A New Standard Archive File for Crystallography. Acta Crystallographica Section A.

[34] Kroenlein K, Löwner R, Wang W, Diky V, Smith T, Muzny CD, Chirico RD, Kazakov A, Sloan ED, Frenkel M Standardization and Software Infrastructure for Gas Hydrate Data Communications.

[35] Adisasmito S, Frank RJ, Sloan ED (1991). Hydrates of Carbon Dioxide and Methane Mixtures. Chemical and Engineering Data.

[36] Udachin KA, Ratcliffe CI, Enright GD, Ripmeester JA (1997). Structure H Hydrate: a Single Crystal Diffraction Study of 2,2-dimethylpentane5(Xe, H2S)-34H20. Supramol Chem.

